# Synchronous Malignant Peripheral Nerve Sheath Tumor and Adenocarcinoma of the Prostate: Case Report and Literature Review

**DOI:** 10.1155/2016/2457416

**Published:** 2016-10-31

**Authors:** Nikolaos Ferakis, Antonios Katsimantas, Konstantinos Bouropoulos, Antonios Farmakis

**Affiliations:** Department of Urology, Korgialenio-Benakio Hellenic Red Cross Hospital, Athanasaki 1, 115 26 Athens, Greece

## Abstract

Malignant Peripheral Nerve Sheath Tumors (MPNSTs) of the prostate are extremely rare. A very unusual case of simultaneous adenocarcinoma and MPNST of the prostate is reported. A 60-year-old Caucasian male presented for annual urologic examination. Digital rectal examination revealed a painless, toughish, and asymmetrically enlarged prostate. Serum prostate-specific antigen was 1 ng/mL. Radiologic examinations demonstrated a large mass, which was arising from the left peripheral lobe of the prostate. The patient underwent transrectal ultrasound-guided biopsy of the prostate which revealed a smooth muscle tumor of uncertain malignant potential. Radical retropubic prostatectomy with en bloc removal of the mass and the seminal vesicles was performed and histology demonstrated low-grade MPNST and adenocarcinoma of the prostate. To the best of our knowledge, this is the first report of simultaneous prostatic adenocarcinoma and MPNST in the English literature.

## 1. Introduction

Malignant Peripheral Nerve Sheath Tumor (MPNST) is a rare soft tissue sarcoma. Sarcomas of the prostate are extremely rare. The aim of this article is to present the first case of simultaneous prostatic adenocarcinoma and MPNST in the English literature.

## 2. Case Presentation

In December 2015, a 60-year-old male patient visited the outpatient clinic of our department for routine urologic examination. Past medical history included hypertension, diabetes mellitus, acute myocardial infarction three years ago, and ischemic cerebrovascular accident thirteen years ago. On digital rectal examination, the prostate was painless, asymmetrically sizable, and toughish, with no other clinical findings. The patient's blood count and blood chemistry levels were in the normal range. Serum PSA was 1 ng/mL.

**Figure 1:**
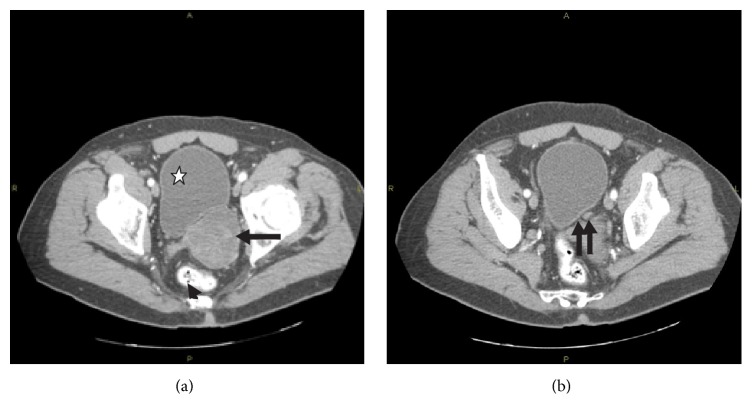
Abdominal/pelvic CT scan image demonstrating (a) a solid, lobed, well-circumscribed mass (arrow) compressing the rectum (arrowhead) and the posterior wall of the urinary bladder (white star) and (b) the distal part of the left ureter (double arrow) between the mass and the bladder wall.

**Figure 2:**
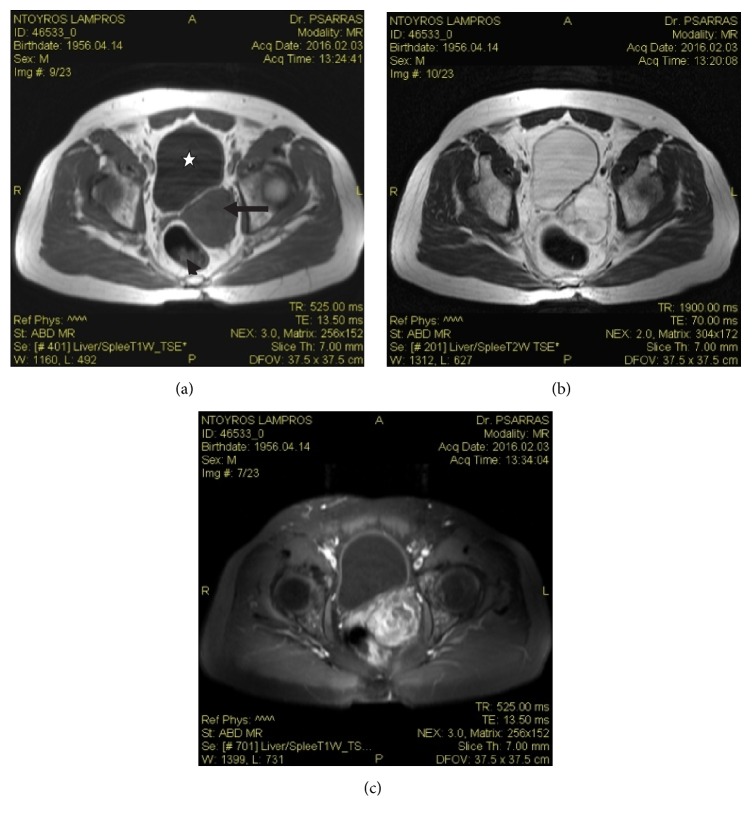
(a) T1-weighted axial MRI image demonstrating a fusiform, lobed, well-circumscribed, hypointense lesion (arrow) with regular borders, compressing the rectum (arrowhead) and the posterior wall of the urinary bladder (white star). (b) T2-weighted axial MRI image showing the lesion having hyperintense signal and containing thin septa. (c) T1-weighted postcontrast axial MRI demonstrating heterogeneous enhancement of the lesion's lobules after intravenous administration of paramagnetic contrast agent.

Transabdominal ultrasonography demonstrated a solid, large mass, in contact with the urinary bladder. The prostate's volume was 31 mL and the postvoid residual of urine was 62 mL. Abdominal/pelvic computed tomography (CT) scan demonstrated the presence of a solid, lobed, well-circumscribed mass, which was arising from the left peripheral zone of the prostate and a possible infiltration of the left seminal vesicle. The urinary bladder wall was normal and the distal part of the left ureter crossed between the mass and the bladder wall (Figure 1). Transrectal ultrasound-guided biopsy of the prostate revealed a smooth muscle tumor with uncertain malignant potential (STUMP). Magnetic resonance imaging (MRI) revealed a fusiform, lobed, well-circumscribed, 6.5 × 6.5 × 6.0 cm in size mass, with regular borders, arising from the left peripheral zone of the prostate. The mass compressed the rectum, the posterior wall of the urinary bladder, and the left seminal vesicle/vas deferens. The lesion had hyperintense signal on T2-weighted sequences and hypointense signal on T1-weighted sequences and contained thin septa. The adipose tissue around the lesion was not infiltrated. There was heterogeneous enhancement of the lesion's lobules after intravenous administration of paramagnetic contrast agent (Figure 2). Chest CT scan and bone scan were negative. The patient underwent 18-fluorodeoxyglucose-positron emission tomography (18-FDG-PET-CT) from the base of the skull to the mid-thigh, which revealed abnormal uptake in the prostatic mass.

The patient underwent radical retropubic prostatectomy with en bloc removal of the mass and the seminal vesicles. A double-J ureteral stent was placed prophylactically in the left ureter, which was removed after the mass excision. The patient had an uneventful postoperative course.

**Figure 3:**
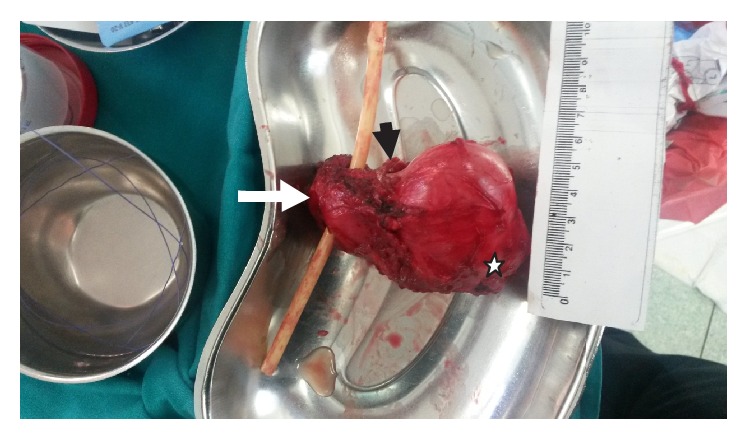
The surgical specimen. The prostate, 5 × 3.8 × 3.5 cm in size (arrow), the right seminal vesicle (arrowhead), and the tumor, 7 × 6.5 × 5.7 cm in size, which invaded the left prostatic lobe (white star).

**Figure 4:**
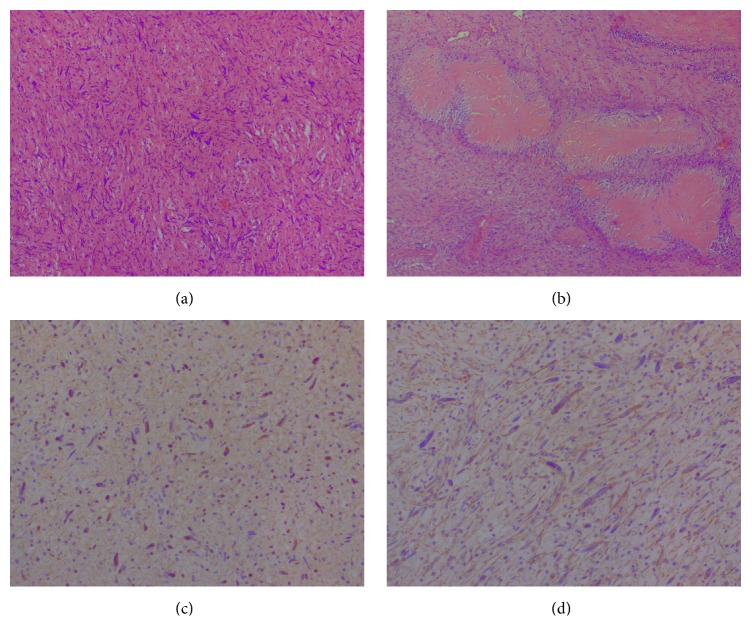
Histological images: (a) intense nuclear atypia (hematoxylin and eosin stain, ×100), (b) Verocay bodies (hematoxylin and eosin stain, ×100), (c) immunohistochemical reaction for S-100, and (d) immunohistochemical reaction for vimentin.

The total resected specimen weighed 145 gr. Macroscopic examination revealed a tan-gray mass, 7 × 6.5 × 5.7 cm in diameter, which invaded the posterior part of the left prostatic lobe (Figure 3). The prostate measured 5 × 3.8 × 3.5 cm. On cut section, the mass had gray-pale yellow color, nodular appearance, and myxoid consistency and infiltrated the left prostatic lobe and the left seminal vesicle. Microscopically, the tumor consisted of spindle cells arranged in bundles or incidentally, with elongated, oval, or thickened, hyperchromatic nuclei, with pleomorphism. The mitotic activity was low, the cellularity was low to mild, and there were a lot of myxoid substances with aggregation of inflammatory cells around the spindle cells. Microcalcifications with ossification and Verocay body formations were identified. The lesion infiltrated the periprostatic adipose tissue. The neoplastic population was immunohistochemically positive for S-100 and vimentin (Figure 4). There were a few cells exhibiting CD34, Bcl-2, and CD56 immunoreactivity. The microscopic examination revealed also a prostatic adenocarcinoma, Gleason Score 6 (3 + 3), confined in the right lobe (pT2a). These findings were consistent with the diagnosis of low-grade MPNST and adenocarcinoma of the prostate.

Abdominal MRI and chest CT scan were normal three months postoperatively and the patient received adjuvant radiotherapy. Serum PSA was 0 ng/mL. The patient has no signs of relapse and is in good general condition 6 months after the operation.

## 3. Discussion

MPNSTs arise from a peripheral nerve or from a preexisting benign nerve sheath tumor or demonstrate Schwann cell differentiation on histology [1–3]. Furthermore, any malignant spindle cell tumor in a patient with neurofibromatosis-1 (NF-1) is considered MPNST, unless proven otherwise [1]. The term MPNST replaces a number of previously used names including malignant schwannoma, neurofibrosarcoma, and neurogenic sarcoma [2, 3].

MPNSTs comprise 5–10% of all soft tissue sarcomas [1, 2]. Sarcomas of the prostate account for 0.7% of all malignant prostatic tumors [4]. Forty percent of MPNSTs are sporadic and the incidence in the general population is 0.001% [1, 3]. The median age for sporadic MPNST is between 30 and 60 years, with no gender predilection [3]. Half of MPNSTs occur in patients with NF-1 [1–3].

MPNSTs may occur anywhere along the course of myelinated nerves, but they commonly appear in or near a nerve of the trunk or the limbs [1, 5]. Pelvic MPNSTs mostly originate from the sacral or the hypogastric plexus [1, 2, 6]. Patients present with an enlarging mass that may cause compression, displacement, or invasion of adjacent structures [1–3, 6]. In most circumstances, the size of the mass is greater than 5 cm at presentation and up to 50% of patients present with metastases, usually in the lung [1–3]. In our case, the patient was 60 years old, without history of NF-1. He had no symptoms, although the size of the mass was greater than 5 cm, and there was no evidence of metastases.

The differential diagnoses include benign neurofibroma, fibrosarcoma, liposarcoma, ganglioneuroma, hydatid cyst, hematoma, and connective tissue diseases [6].

Preoperative radiologic examinations play a vital role in the diagnosis of a MPNST and in surgical planning. Ultrasonography can discriminate solid tumors from cystic masses [7]. CT scan shows well-defined, low, or mixed attenuation masses with cystic necrotic central areas [7]. Hemorrhage, calcification, and hyalinization may be present, but all these changes are not specific and the main use of CT is for detection of metastases [1, 2, 6, 7]. MRI is the modality of choice for characterizing the anatomical extent of the tumor for surgical planning and helps differentiate MPNSTs from benign plexiform neurofibromas [1–3]. The lesion is usually fusiform with tapered ends and is oriented longitudinally along the direction of a peripheral nerve [1, 2]. Fat suppression sequences may allow better visualization of the nerve(s) involved [1]. 18-FDG-PET-CT scan helps in differentiating MPNST from benign neurofibroma in NF-1 patients and detecting malignant transformation of benign plexiform neurofibromas [1, 3]. Imaging criteria are generally considered unreliable in differentiating MPNST from a benign schwannoma. Big irregular lesion, with rapid growth on interval imaging, heterogeneity, invasion of fat planes, and edema surrounding the lesion favor MPNST diagnosis [2].

Macroscopically, MPNSTs are globoid or fusiform in shape, fleshy, and firm to hard in consistency and their color is typically tan-gray on cut section [1]. Areas of necrosis or cyst formation are commonly present and the lesions may be covered by a fibrous pseudocapsule and invade surrounding soft tissue, as in our case [1, 7].

Microscopically, the tumor is characterized by hypercellular fascicles of spindle cells interrupted by hypocellular myxoid areas, often with hypercellular areas localized in close proximity to blood vessels [1, 3, 6–8]. The spindle cells are relatively large, with long, hyperchromatic, wavy, or “serpentine” nuclei [1, 8]. Malignancy is usually suggested if high mitotic activity, increased cellularity, pleomorphism, nuclear atypia, blood vessel infiltration, and tumor necrosis are shown histologically [1–3, 6]. Heterologous elements, such as skeletal muscle, bone, and cartilage, are present in approximately 15% of tumors and may portend an even poorer prognosis [3, 8]. There is no pathognomonic immunohistochemical study for MPNST [1, 3]. S-100, which is traditionally regarded as the best marker for MPNST, was positive in our case. However, it has limited diagnostic utility and is positive in about 50–90% of the tumors [2, 8]. Leu-7 and myelin basic protein are noted in 50% and 40% of cases, respectively [1, 2]. In general, a combination of antigens is used to help exclude other spindle cell lesions and confirm the diagnosis of MPNST [2]. In our case, vimentin, CD34, Bcl-2, and CD56 were only partially positive.

Complete surgical extirpation of the tumor with clear margins is the treatment of choice [1–3, 5–8]. It may be necessary to sacrifice adjacent tissue and viscera [7]. Furthermore, it has been shown that, in case of malignancy, the local recurrence rate after marginal excision is 72% versus 11.7% after wide margin resection [6]. Therefore, it is highly recommended to send a biopsy for frozen section before choosing the surgical approach [6]. The tumor in our case was infiltrating the left prostatic lobe and the left seminal vesicle. A complete excision was only possible with en bloc removal of the mass with the prostate and seminal vesicles. According to the literature, it is unknown if the location of the tumor has a prognostic value [1]. Adjuvant radiation therapy was found to improve local control and reduce local recurrence rates in many series, but most of series have found no benefit with respect to overall survival [1–3]. Adjuvant chemotherapy has not been proven to significantly improve survival and is often considered for patients with unresectable tumors or metastatic disease [1].

Prognosis is poor. Five-year overall survival is reported to be 15–50% [3]. The local and distant recurrence rate have been reported to range from 40 to 65% and from 40 to 68%, respectively [2]. Longer survival has been correlated with complete surgical excision, no local recurrence, small tumor size (<5 cm), and low histological grade [1–3]. In our patient, the tumor was 7 cm in biggest diameter and the tumor aggressiveness was low. Furthermore, a simultaneous low risk prostatic adenocarcinoma was present, and, as mentioned, it is the first case reported in the literature. No genetic pathways have been reported, predisposing to simultaneous occurrence of the two malignancies, neither is it known if the prognosis of MPNST could be affected, especially in the presence of a low risk prostatic adenocarcinoma.

Follow-up guidelines have not been defined, but many authors recommend MRI imaging every 3 months for detection of local or distant recurrence [1].

Although there has been progress in diagnosis of MPNSTs, related with advances in imaging methods, there is still a lot to be researched with regard to the genetics and the molecular biology of these tumors [2]. Defining characteristics on a molecular level might allow for earlier disease detection, more effective targeted chemotherapy, and more reliable prognostic information [2].
